# *Streptococcus pneumoniae* Serotypes Carried by Young Children and Their Association With Acute Otitis Media During the Period 2016–2019

**DOI:** 10.3389/fped.2021.664083

**Published:** 2021-07-05

**Authors:** Esra Ekinci, Stefanie Desmet, Liesbet Van Heirstraeten, Colette Mertens, Ine Wouters, Philippe Beutels, Jan Verhaegen, Surbhi Malhotra-Kumar, Heidi Theeten, NPcarriage Group

**Affiliations:** ^1^Centre for Evaluation of Vaccination, Vaccine and Infectious Disease Institute, University of Antwerp, Antwerp, Belgium; ^2^Reference Centre for Pneumococci, University Hospitals Leuven, Leuven, Belgium; ^3^Laboratory of Medical Microbiology, Vaccine and Infectious Disease Institute, University of Antwerp, Antwerp, Belgium; ^4^Centre for Health Economics Research and Modelling Infectious Diseases, University of Antwerp, Antwerp, Belgium

**Keywords:** pneumococcal acute otitis media, serotypes, children, pneumococcal conjugate vaccine, AOM, PCV, *Streptococcus pneumoniae*

## Abstract

**Background:** *Streptococcus pneumoniae* (Sp) is a major cause of acute otitis media (AOM). Pneumococcal conjugate vaccine (PCV) programs have altered pneumococcal serotype epidemiology in disease and carriage. In this study, we used samples collected during a cross-sectional study to examine if the clinical picture of acute otitis media (AOM) in young children exposed to the PCV program in Belgium was related to the carried pneumococcal strains, and if their carriage profile differed from healthy children attending daycare centers.

**Material/Methods:** In three collection periods from February 2016 to May 2018, nasopharyngeal swabs and background characteristics were collected from children aged 6–30 months either presenting at their physician with AOM (AOM-group) or healthy and attending day care (DCC-group). Clinical signs of AOM episodes and treatment schedule were registered by the physicians. Sp was detected, quantified, and characterized using both conventional culture analysis and real-time PCR analysis.

**Results:** Among 3,264 collected samples, overall pneumococcal carriage and density were found at similar rates in both AOM and DCC. As expected non-vaccine serotypes were most frequent: 23B (AOM: 12.3%; DCC: 17.4%), 11A (AOM: 7.5%; DCC: 7.4%) and 15B (AOM: 7.5%; DCC: 7.1%). Serotypes 3, 6C, 7B, 9N, 12F, 17F, and 29 were more often found in AOM than in DCC (*p*-value < 0.05), whereas 23A and 23B were less often present in AOM (*p*-value < 0.05). Antibiotic non-susceptibility of Sp strains was similar in both groups. No predictors of AOM severity were identified.

**Conclusion:** In the present study, overall carriage prevalence and density of *S. pneumoniae* were found similar in young children with AOM and in healthy children attending day-care centers in Belgium. Certain serotypes not currently included in the PCV vaccines were found to be carried more often in children with AOM than in DCC, a finding that might suggest a relationship between these serotypes and AOM.

## Introduction

Acute otitis media (AOM) is one of the most common pediatric infections and has been described as one of the leading causes of antibiotic prescription in children in industrialized countries (1, 2). It is estimated that 80-90% of children have an episode of acute otitis media (AOM) before the age of three, with a peak incidence between 6 and 15 months (3, 4).

*Streptococcus pneumoniae* (Sp) is the most common OM pathogen and has been associated with first or early otitis media episodes, more severe AOM signs and symptoms, and potential middle-ear damage (5). It colonizes the nasopharynx within the first few weeks or months of life, and carriage is a highly dynamic process (6, 7). Sp is however commonly a quiescent colonizer of the upper respiratory tract (URT) that only becomes pathogenic when changes in the upper respiratory tract allow the organism to achieve a pathogenic inoculum that overcomes innate and adaptive host defense. Capacity to colonize and invade, differs and it is related to the capsular serotype as well as to the history of colonization with this serotype. So far, at least 100 distinct capsular serotypes have been described (8, 9). Inactivated pneumococcal conjugate vaccines (PCV) were developed to prevent invasive disease caused by the most pathogenic serotypes. Before PCV introduction, the most common pneumococcal serotypes causing AOM worldwide were 3, 6A, 6B, 9V, 14, 19A, 19F and 23F (10–13). Administration of PCV has also led to a reduction of AOM caused by Sp (3, 14).

Since PCV implementation in children, a tremendous decrease in vaccine serotype related carriage and invasive pneumococcal disease cases have been observed in countries with a high vaccine uptake (15). However, PCVs only target a limited number of frequently invasive serotypes. Furthermore, the protection against the vaccine serotypes opens new nasopharyngeal niches for colonization, which favors conditions for serotype replacement. Hence, PCV introduction induced significant changes worldwide in the epidemiology of Sp in disease, carriage and antimicrobial (non-)susceptibility. Belgium has a unique pneumococcal vaccine program history: serotype coverage moved from PCV7, implemented in 2007 to PCV13 in 2011 and from PCV13 to PCV10 in 2016. This switch happened because both PCV vaccines were considered equivalent by the National Immunization Technical Advisory Group (NITAG) at that time, based on invasive disease serotype epidemiology, and thus the outcome of the regular tender process (after expiry of the contract) was mainly guided by logistic and financial factors (16). At every change of vaccine brand, a cohort of children received mixed schedules. Although it was recommended to use the same vaccine for the primary doses in the first year of life, these vaccines were not automatically reserved per child and thus children received all kinds of mixed schedules with a total number of three PCV-doses. Monitoring nasopharyngeal carriage to complement invasive disease data started at the time of the switch to PCV10, and involved two populations of young children in whom pneumococcal carriage was known to be high, namely children attending daycare centers and children suffering from AOM (17).

No recent data were available on the serotype distribution and antibiotic susceptibility of pneumococcal isolates in Belgian children with AOM, and their relationship with clinical symptoms. The carriage study investigating them simultaneously with healthy infants attending daycare centers within a single study protocol created an opportunity to investigate whether there was a difference in serotype distribution, carriage density or antimicrobial non-susceptibility between children suffering from AOM and healthy children attending DCC. It also allowed to examine whether there is a link between the clinical severity of the AOM and the carried serotypes.

## Materials and Methods

The data were collected during an ongoing study that monitors nasopharyngeal carriage in infants in Belgium since 2016, after the introduction of PCV10 in the PCV program. In the first 3 years, this study investigated nasopharyngeal carriage in two infant populations with high reported carriage of Sp, namely healthy infants attending DCC and infants suffering from AOM. The study protocol was described in detail by Wouters et al. (17, 18) and is summarized here. The study was in line with the Declaration of Helsinki, as revised in 2013. Approval to conduct the current study with ID 15/45/471 was obtained from the University of Antwerp and University Hospital of Antwerp ethics committee (Commissie voor Medische Ethiek van UZA/UA) on 30 November 2015.

The recruitment was restricted to non-summer seasons (November to end May) from 2016 to 2018. In period 1, sampling was performed between January and June 2016, i.e., during and shortly after the switch from PCV13 to PCV10 in the Belgian vaccination program, while in period 2 (2016–2017) and period 3 (2017–2018) samples were collected between November and May. Randomly selected DCCs and hospitals/ pediatric outpatient services were located over the three Belgian regions (the Flemish Region, the Walloon Region and the Brussels-Capital Region). Age limits for inclusion in both populations were 6 months and 30 months. In the first recruitment season, region-specific age criteria were added to include only infants who were offered PCV13 for both primary vaccine doses in the first year of life. AOM was defined explicitly as the acute onset of symptoms (within the preceding seven days). The clinical inclusion criteria were ear tugging, fever, crying, irritability, difficult sleeping, diminished activity and/or appetite together with otoscopic signs. Otoscopic signs are including tympanic opacity due to middle ear effusion combined with moderate or intense bulging, or slight bulging accompanied with either recent onset of otalgia/ear tugging or marked unilateral erythema of the membrane or new onset otorrhea (including through tympanic tube). Exclusion criteria for AOM infants as well as healthy infants in DCC were (1) use of antibiotics in the past seven days, (2) presence of a chronic and severe associated pathology and (3) previous inclusion in the study within the same collection period. AOM infants who were recruited in hospitals (outpatient services) had extra exclusion criteria: (1) referral by a GP or (2) having received three or more antibiotic treatments in the past 3 months.

### Nasopharyngeal Samples (NP) and Questionnaire

Parents consenting to participate received a questionnaire regarding their infant's demographics and clinical characteristics, and pneumococcal vaccination status. A trained nurse or a general practitioner/pediatrician collected a single nasopharyngeal (NP) sample with a flocked nylon swab in 1 mL STGG (skim milk, tryptone, glucose and glycerol). Signs of common cold in DCC infants were defined as coughing and/or running nose at sampling.

The samples were stored frozen and assessed by culture at the National Reference Center for Pneumococci (University Hospitals in Leuven) and RT-PCR analysis at the Laboratory of Medical Microbiology (University of Antwerp). Cultured Sp strains were serotyped by performing Quellung-reaction and antimicrobial susceptibility to penicillin, tetracycline, erythromycin, levofloxacin and cotrimoxazole (the most frequently used antibiotics in respiratory disease over all ages) was determined by disc diffusion following CLSI 2016 guidelines. If non-susceptibility for levofloxacin or penicillin was identified, the minimal inhibitory concentration (MIC) was determined. A MIC of >2 mg/L and >0.06 mg/L was interpreted as levofloxacin and penicillin non-susceptible, respectively. Quantitative Taqman real-time PCR (qRT-PCR) targeting *lytA*, using StepOnePlus^TM^ Real-Time PCR System (Applied Biosystems ^TM^) was performed to screen the samples for presence of Sp. Bacterial densities were determined based on a standard curve that was set up using 10-fold serially diluted *lytA* PCR product of Sp strain ATCC 49619. Samples and standard curves were run in triplicate. Samples were considered positive for Sp if either culture or PCR (cut off cycle threshold value ≤ 35.0) was Sp-positive (18). *Lyt*A positive samples were pooled and screened for presence of PCV13-non-PCV10 serotypes (3, 6A, 19A) (2).

### Statistical Analyses

All descriptives and statistical analyses were calculated in JMP Pro 14.3. To test for significant differences in proportions the Chi-Square Test (Chi2), or Fisher's Exact Test (FET) where appropriate, were used. To compare overall association between vaccination status and health status, bivariate logistic regression was performed using period and vaccination status as factors and AOM/DCC as outcome. To compare parameters that were not normally distributed the Mann-Whitney U Test (MWU) or Kruskal-Wallis Test (KW) were used as appropriate.

The sample size of the main study was calculated according with its primary aim to compare carriage prevalence of three pneumococcal vaccine serotypes over the first 3 years (2016–2018). For the current study, nasopharyngeal carriage of Sp in infants with AOM and in infants attending DCC, were compared using a *post-hoc* power analysis using R package power.

## Results

### Recruitment and Infant Characteristics

Over the 3-year study period, a total of 3,264 nasopharyngeal samples were collected of which 3,175 were included in the analysis (Table 1). Nasopharyngeal samples of 74 DCC children and 15 AOM children were excluded due to protocol violation. Moreover, four children with AOM and with incomplete questionnaires were excluded from the clinical analysis regarding AOM symptoms. A random selection of 194 DCC-samples collected in 2016–2017 was analyzed only by culture and not by PCR due to restriction at laboratory level.

**Table 1:** Number of included samples, DCCs and clinics per sampling period.

	**Period 1**	**Period 2**	**Period 3**
	**2016**	**2016–2017**	**2017–2018**
DCC setting	85	112	102
DCC samples	769	1096	953
AOM setting	12	21	19
AOM samples	39	122	205

A *post-hoc* power calculation was performed taking the total DCC (*N* = 2809) and AOM (*N* = 366) sample size into account. The sample size allows for the detection of a 15% difference in carriage prevalence of pneumococcal serotypes between healthy DCC infants and AOM infants with 77% power at a significance level of 5% (two-sided).

AOM children differed in several demographic and clinical characteristics from DCC children (Table 2). Overall, children with AOM tended to be younger than children recruited in DCCs. The convenience recruitment of AOM children resulted in a disproportionate regional distribution, which was not the case for DCC children. Overall, breastfeeding for more than 6 months was more frequent in DCC children and parental smoking was more frequent in AOM children. Children with AOM more often had a history of previous hospitalization than DCC children, and they more frequently had siblings. A significantly higher proportion of AOM children had a history of one or more previous AOM episodes over the 3-year study period compared to DCC children. Lastly, antibiotic use in the past 3 months was more frequent in DCC children than AOM children.

**Table 2:** Overall demographic and clinical characteristics of children with AOM and children attending DCC.

		**Children with AOM**		**Children attending DCC**		**Statistical significance**
		***N*** **=** **366**		***N*** **=** **2809**		**(*p*-value for AOM vs. DCC)**
		***n***	***%***	***n***	***%***	
Region	Flanders	197	*53.8*	1489	*53.0*	**0.007**
	Wallonia	141	*38.5*	950	*33.8*	
	Brussels	28	*7.7*	370	*13.2*	
Age (months)	6-12	163	*45.0*	578	*20.6*	** <0.001**
	13-24	154	*42.5*	1497	*53.3*	
	25-30	45	*12.4*	734	*26.1*	
Gender	Male	186	*51.4*	1421	*50.6*	0.786
Preterm delivery	Yes	25	*6.9*	223	*8.0*	0.480
Breastfeeding >6 months	Yes	81	*22.5*	927	*33.1*	** <0.001**
At least one parent smokes	Yes	103	*28.6*	586	*21.0*	** <0.001**
Previous hospitalization	Yes	108	*30.0*	514	*18.5*	** <0.001**
Number of siblings	0	117	*32.6*	1038	*37.5*	** <0.001**
	1	121	*33.7*	1242	*44.9*	
	>1	121	*33.7*	488	*17.6*	
AOM-history	0	101	*35.7*	1994	*73.1*	** <0.001**
	1	86	*30.4*	279	*10.2*	
	>1	96	*33.9*	454	*16.7*	
AB <3 months	Yes	81	*22.6*	777	*29.4*	**0.007**

*Chi-square p-value for statistical significance of AOM vs. DCC. The categorical variables were age (6–12, 13–24, and 25–30 moths), number of siblings (0, 1, >1) and a history of AOM (0, 1, >1). AOM, acute otitis media; DCC, day care center; AB, antibiotics*.

*Percentages are noted in italic and significant p-values are noted in bold*.

**Figure 1:**
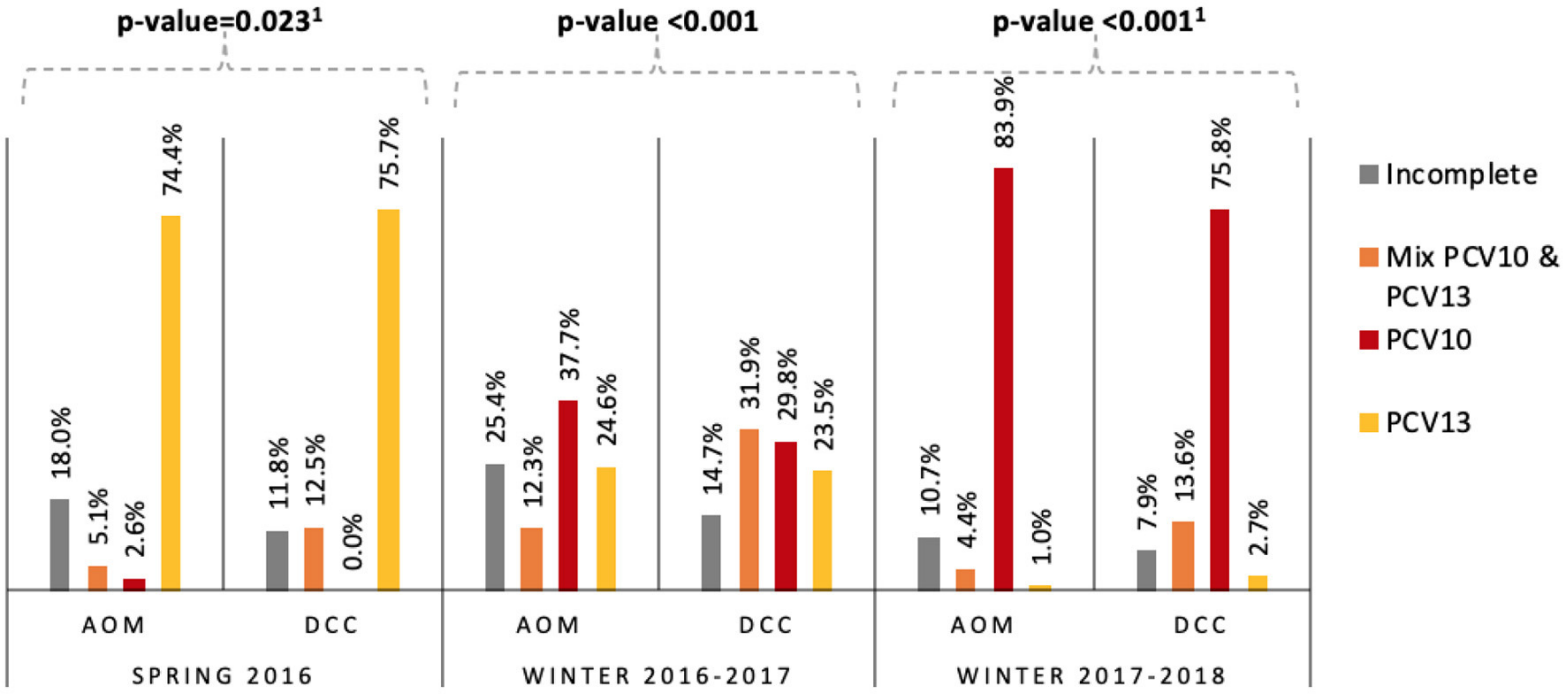
Vaccination status of children with AOM and children attending DCCs per season in Belgium in 2016–2018. Per season, the Chi-square or Fisher's Exact Test p-values for statistical significance of AOM vs. DCC are noted above. ^1^Fishers Exact Test. AOM = acute otitis media, DCC = day care center, PCV10 = age-appropriately vaccinated with 10-valent pneumococcal conjugate vaccine, PCV13 = age-appropriately vaccinated with 13-valent pneumococcal conjugate vaccine; Mix PCV10&PCV13 = age-appropriately vaccinated with a schedule combining both vaccines; incomplete = not vaccinated age-appropriately.

The PCV10-vaccinated proportion increased over the study period from 2.6 to 83.9% and from 0.0 to 75.8%, in AOM children and DCC children respectively. The proportion of PCV13-vaccinated children decreased from 74.4 to 1.0% in AOM and 75.7 to 2.7% in DCCs (Figure 1). In each period the overall difference in vaccination status (as defined in Figure 1) between AOM and DCC was significant.

### Carriage Prevalence Was Similar in AOM Children and in DCC Children

Pneumococcal carriage prevalence was similar in AOM children and in DCC children (AOM: 79.2%; DCC: 77.5%; *p*-value = 0.454), and also among different age categories, for both AOM (6–12 m: 76.7%, 13–24 m: 82.5%, 25–30 m: 77.8%; *p*-value = 0.431) and DCC (6–12 m: 75.8%, 13–24 m: 79.1%, 25–30 m: 75.6%; *p*-value = 0.098).

### Serotype Distribution Slightly Differed Between AOM Children and DCC Children

A total of 2241 Sp strains were cultured and 51 different serotypes were identified. In AOM children, 34 serotypes in 268 strains were identified and in DCC children 49 serotypes in 1973 strains. PCV13 serotypes increased over time in AOM (Y1: 0.0%, Y2: 8.4%, Y3: 8.3%) and DCC (Y1: 1.8%, Y2: 2.6%, Y3: 9.8%), mainly because of an increased prevalence of serotype 19A (AOM: Y1: 0.0%, Y2: 3.6%, Y3: 6.4%; DCC: Y1: 0.6%, Y2: 1.9%, Y3: 8.8%). Serotype 23B, a non-vaccine type, was most frequent in AOM (12.3%) and DCC (17.4%), followed by 11A (7.5%), 15B (7.5%) and 6C (6.3%) in AOM and 23A (8.0%), 11A (7.4%) and 15B (7.1%) in DCC. Serotypes 8 and 19B were found in AOM children (both 0.4%), but not in DCC children. For 9 serotypes, frequency differed significantly between AOM and DCC (Table 3), although the difference was not significant in every separate period. Frequency of vaccine serotypes over time is summarized in Table 8: Appendix 3 in Supplementary Material.

**Table 3:** Difference in serotype distribution of *S. pneumoniae* between children with AOM and children attending DCC in Belgium in 2016–2018.

	**AOM frequency**	**DCC frequency**	**Statistical significance**
	**(%)**	**(%)**	**(*p*-value for AOM vs. DCC)**
3	2.6	0.8	0.015^a^
6C	6.3	3.4	0.030^b^
7B	0.8	0.1	0.039^a^
9N	2.2	0.9	0.048^a^
12F	2.2	0.8	0.032^a^
17F	2.6	0.6	0.003^a^
23A	4.1	8.0	0.025^b^
23B	12.3	17.4	0.036^b^
29	1.1	0.2	0.041^b^

*Only serotypes that were found significantly more in one of the two groups are shown*.

a *Fisher's Exact Test*,

b *Chi2, AOM, acute otitis media; DCC, day care center*.

### Carriage Density of Sp Positive Samples Was Similar in AOM Children and DCC Children

The overall median pneumococcal DNA density in AOM-infants was 5.0 × 10^5^ copies/μl (3.3 × 10^5^-7.6 × 10^5^ copies/μl) and in DCC-infants 4.2 × 10^5^ copies/μl (3.8 × 10^5^-5.7 × 10^5^ copies/μl) (MWU for median; *p*-value = 0.154). In an age-specific analysis, median density was higher in carriers with AOM in the 6–12 months and 25–30 months age category compared to carriers in DCC in the same age categories (Table 4).

**Table 4:** Median *S. pneumoniae* carriage density, expressed in × 10^5^ copies/μl, per age group for AOM and DCC in 2016–2018.

	**6–12 months**	**13–24 months**	**25–30 months**
AOM	3.90	4.78	9.47
DCC	2.53	5.20	3.33
Statistical significance (*p*-value MWU for AOM vs. DCC)	**0.035**	0.819	**0.045**

*AOM: 6–12 months N = 117, 13–24 months N = 115, 25–30 months N = 30; DCC: 6–12 months N = 366, 13–24 months N = 1027, 25–30 months N = 489). AOM, acute otitis media; DCC, day care centers; MWU, Mann-Whitney U-Test*.

*significant p-values are noted in bold*.

### Antimicrobial Non-susceptibility Was Similar in AOM Children and DCC Children

In the AOM population, 19A strains were more frequently non-susceptible to penicillin compared to the DCC population (AOM: 8.8%; DCC: 1.2%; *p*-value = 0.002). The susceptibility profile of other serotypes was similar in both populations.

Over time, non-susceptibility of Sp strains to more than one antibiotic increased borderline non-significantly in the AOM population (Y1: 18.5%, Y2: 25.3%, Y3: 35.7%; p-value = 0.092) and increased significantly in the DCC population (Y1: 18.4%, Y2: 26.3%, Y3: 30.5%; *p*-value < 0.001).

### Clinical Signs in Children With AOM Were Not Associated With Vaccine Type Carriage

Clinical signs were analyzed to look for differences between AOM infants carrying vaccine serotypes and non-vaccine serotypes. Otoscopic signs are summarized in Table 5. The average temperature was 39.1°C (36.0–41.6°C). A small number of patients had no fever (8.7%, 30/347), while 47.8% (166/347) had a temperature of >39°C.

**Table 5:** Clinical spectrum of acute otitis media.

	**None**	**Unilateral**	**Bilateral**
	**% (*n*)**	**% (*n*)**	**% (*n*)**
Otorrhea	85.2 (304/357)	11.2 (40/357)	3.6 (13/357)
Effusion	20.2 (71/352)	33.8 (119/352)	46.0 (162/352)
Bulging	10.3 (37/358)	71.0 (254/358)	18.7 (67/358)

*Missing values (otorrhea: 5, effusion: 10, bulging: 4) were due to incomplete questionnaires*.

Median recovery time was 4 days; 47.5% (124/261) of children recovered within 3 days, and 8.8% (23/261) of children needed more than 8 days. A second visit for AOM within 1 month was reported for 18% (65/362) of the children. Reasons for a second visit were: deterioration established by a physician (9.2%, 6/65), deterioration according to the parents during a follow-up phone call (10.8%, 7/65), additional infection established by a physician (24.6%, 16/65), relapse according to the parents during a follow-up phone call (35.4%, 23/65) or unknown reasons (20.0%, 13/65).

No differences in clinical signs were observed between children carrying vaccine serotypes and non-vaccine serotypes, except for antibiotic use. Antibiotic prescription occurred in 77.3% of all cases (279/361, 1 missing value). Children carrying non-vaccine serotypes (82.3%, 191/232) more often received antibiotics than children carrying vaccine serotypes (64.3%, 18/28) (*p*-value = 0.023).

## Discussion

In this 3 year nasopharyngeal carriage study, differences were identified in Sp serotype distribution, but not in carriage density or antimicrobial non-susceptibility between children with AOM and healthy children attending DCC.

Overall Sp carriage prevalence was similar and high in the two populations (AOM: 79.2%; DCC: 77.5%; *p*-value = 0.454). Carriage prevalence of Sp vary between studies from 11.8 to 71.2% in PCV vaccinated AOM children and from 8.3 to 89.5% in healthy PCV vaccinated children (18–21). The carriage prevalence in the current study is at the higher end of the reported range as in this study two child populations that are known to have a higher pneumococcal carriage prevalence were selected (22–25).

Serotypes 3, 6C, 9N, 12F, 17F, 7B, and 29 were found more frequently in AOM children than in DCC, although they were all together uncommon (AOM: 17.9%; DCC: 6.7%). Serotype 3 was thus the only vaccine serotype that was identified significantly more frequently in children with AOM than in children attending DCC. This is in line with previous findings that associated serotype 3 to a high invasive capacity and more-severe disease (26–30). Serotypes 9N and 12F have not previously been associated with AOM, though they have been associated with invasive pneumococcal disease (IPD) (27, 29, 31–33). Serotype 17F has previously been associated with serious clinical outcomes and higher case-fatality, but not with AOM (34, 35).

However, as the prevalence of PCV13 vaccine serotypes increased over the 3 year study period, serotype 19A was the most common PCV13 vaccine serotype in the third period of our study for both populations. Serotype 19A was also the most commonly identified serotype in IPD (27%) and middle ear fluid (MEF) (26%) in 2018 in Belgian children (36). In other studies looking at clinical manifestation of AOM episodes by serotype, serotype 19A was (one of) the most frequent serotype(s) identified in MEF (36–39), and 19A was also found among the most frequently carried serotypes in children with AOM in other nasopharyngeal carriage studies (38, 40). After PCV10 introduction in Bulgaria, the prevalence of 19A in the MEF of children diagnosed with AOM was 5.6% (41). This is similar as the nasopharyngeal carriage prevalence of serotype 19A (6.4%) that we found in children with AOM 2–3 years after PCV10 introduction in Belgium. Serotype 19A has been reported as a serotype that is frequently non-susceptible to antibiotics, especially after PCV7 or PCV10 introduction (10, 42, 43). After PCV7 introduction in France, penicillin non-susceptibility for serotype 19A in children with AOM ranged from 77.6 to 92.0% (40). However, in the present study non-susceptibility to penicillin of serotype 19A strains was a lot lower (AOM: 8.8%; DCC: 1.2%). Since children who were treated with oral antibiotics in the seven days prior to sampling were excluded, some non-susceptible strains could have been missed. In the current study, non-susceptibility to penicillin was investigated and overall no difference was found in non-susceptibility to penicillin in the AOM group compared to the DCC group. The current guideline for antibiotic use for AOM can be maintained as non-susceptibility to penicillin is still limited, but if this should increase as a result of changes in the serotype prevalence, then this needs to be reconsidered.

The PCV7 serotype 19F also continues to circulate and was found in children with AOM in all periods, as was the case in other PCV7-, PCV10- and PCV13-administering countries (10, 30, 37, 44–49). Among non-PCV13 serotypes, serotype 23B was most prevalent in both groups of children, and has also been reported as the most common colonizer in other studies (10, 30, 45). Other studies showed that serotypes 23A and 23B are carried for a longer period and therefore are detected more frequently (28). In the current study, 23A and 23B were found more frequently in healthy children attending DCC than in children with AOM.

No significant difference in carriage density of Sp between children with AOM and children attending DCC was found. A higher density was expected in children with AOM, since a higher pneumococcal load has been found associated with acute respiratory disease (50–52). The study may be underpowered, as in period three, when the highest number of children with AOM were recruited, the difference in density was borderline non-significant with a higher value in AOM (AOM: 5.3 × 10^5^; DCC: 3.3 × 10^5^; *p*-value = 0.086). A possible explanation is that Sp transmission is high in Belgian DCCs, as high density of Sp in the nasopharynx is suggested to be a predictor of both transmission and disease probability (53, 54). Dunne et al. found that having upper respiratory tract infection symptoms was associated with an increased pneumococcal density (55–57). In the current study, 36.4% of children in DCCs presented common cold symptoms, which associates with the high pneumococcal density in DCC-children. For children aged between 25 and 30-months, a three times higher carriage density was observed in the AOM-population compared to the DCC-population. Since risk of AOM development decreases with age (58), this finding suggests a role of carriage density in disease development.

In the current study, we compared children carrying vaccine serotypes and non-vaccine serotypes, and did not find a difference for any of the mentioned symptoms, neither in a separate analysis nor using a summarizing score that combined the different elements of the clinical evaluation by the treating physician (Table 9: Appendix 4 in Supplementary Material). This can be due to the low number of vaccine type carriers in this study that do not allow to detect a clear difference in AOM children carrying vaccine serotypes and non-vaccine serotypes.

A major strength of this study is the combined use of culture and molecular methods to explore pneumococcal carriage in nasopharyngeal microbiota. In addition, this study started after a switch from PCV13 to PCV10, which has rarely occurred worldwide and with different preceding PCV program history. Another strength is the that the two populations, children with AOM and healthy carriers, were recruited simultaneously within the same study protocol and using the same methods. However, some limitations should be considered when interpreting the presented results. First, in children with AOM we cannot be sure that the carried Sp serotype was the cause of the AOM episode, especially since 91.4 and 95.5% of Sp positive samples also carried *Haemophilus influenzae* and *Moraxella catarrhalis* respectively. MEF samples allow for more accurate identification of the causative agent, but are not standard practice in first-line AOM approach in Belgium. Secondly, findings refer to the specific child populations that were studied and cannot be generalized to non-responsive or chronic otitis media, or to children of all ages. In addition, 148 DCC children representing 303 isolates participated more than once, but in different study periods. A sensitivity analysis that only used their first sample confirmed all findings for child characteristics, carriage prevalence and carriage density. Finally, any trends in time should be interpreted cautiously. Study population characteristics were not constant over time, especially in the AOM population in whom recruitment was more successful toward the end of the study. Moreover, calendar time was different in the first collection period than in the later ones, which might have exaggerated season-dependent changes in serotype distribution. However, the marked 19A increase was the only time trend we identified. A trend we suppose not to be due to differences in season as no season-dependency of 19A carriage has been reported so far. After the PCV13-to-PCV10 switch, the carriage proportion of PCV13-non-PCV10 vaccine serotype 19A showed an increasing trend in healthy children attending day care centers (DCC) (2). This increasing trend of serotype 19A was also observed in Belgian children with invasive pneumococcal disease (59). Consequently, PCV13 has been implemented again in 2019.

We can conclude that the change in the Belgian PCV program has induced similar changes in both AOM and DCC infants, with an increase of PCV13 vaccine serotype 19A from 0.0% in year 1 to 6.4% in year 3 for the AOM population and from 0.6% in year 1 to 8.8% in year 3 for the DCC population. This study shows that young children with AOM did not carry *S. pneumoniae* more frequently or at higher load than healthy children attending DCC. Nevertheless, the serotypes that were found more common in AOM children were mainly non-vaccine serotypes. Although an interesting finding, it should be taken with caution as only nasopharyngeal swabs were collected in the present study, and it is known that results from nasopharyngeal swabs and MEF swabs in AOM patients are not fully consistent. Lastly, no differences were observed in the clinical outcome of AOM in children carrying vaccine types compared to children carrying non-vaccine types.

## Data Availability Statement

The raw data supporting the conclusions of this article will be made available by the authors, without undue reservation.

## Ethics Statement

Approval to conduct the current study with ID 15/45/471 was obtained from the University of Antwerp and University Hospital of Antwerp ethics committee (Commissie voor Medische Ethiek van UZA/UA). Parental consent was obtained.

## NPcarriage Study Group

David Tuerlinckx, CHU Dinant-Godinne, Université Catholique de Louvain, Yvoir, Belgium; Adam Finn, University of Bristol, School of Cellular and Molecular Medicine, Bristol, UK; Koen Van Herck, Department of Public Health and Primary Care, Ghent University, Gent, Belgium, Center for the Evaluation of Vaccination, Vaccine and Infectious Disease Institute, University of Antwerp, Wilrijk, Belgium; Robert Cohen, Université Paris Est, IMRB-GRC GEMINI, 94000 Créteil, France; Christine Lammens, Laboratory of Medical Microbiology, Vaccine and Infectious Disease Institute, University of Antwerp, Wilrijk, Belgium; Marc Verghote (Private pediatrician); Marc De Meulemeester (Private pediatrician); Barbara De Wilde (Private pediatrician); Kate Sauer, Pediatric Department, AZ Sint-Jan, Brugge, Belgium; Luc Pattyn, Pediatric Department, AZ Turnhout, Belgium; Bart Rutteman, Pediatric Department, AZ Sint-Blasius, Dendermonde, Belgium; Caroline Genin, Pediatric Department, C.H.C Espérance, Saint-Nicolas, Belgium; Diane Stroobant, Pediatric Department, Grand Hôpital de Charleroi, Charleroi, Belgium; Ilse Ryckaert, Pediatric Department AZ-Nikolaas, Sint-Niklaas, Belgium; Tessa Goetghebuer, Pediatric Department, CHU Saint-Pierre, Brussels, Belgium; Zornitsa Vassileva, Pediatric Department, AZ Lokeren, Lokeren, Belgium.

## Author Contributions

All authors listed have made a substantial, direct and intellectual contribution to the work, and approved it for publication.

## Conflict of Interest

The authors declare that the research was conducted in the absence of any commercial or financial relationships that could be construed as a potential conflict of interest.
