# Supplementary material for: Streptococcus pneumoniae Serotypes Carried by Young Children and Their Association With Acute Otitis Media During the Period 2016–2019
Source: Front Pediatr. 2021 Jul 5;9:664083. doi: 10.3389/fped.2021.664083 (PMC8286995; doi:10.3389/fped.2021.664083)
Supplement: Supplementary file 1 [file Table_1.docx]

**Appendices**

**Appendix 1**

|  | | Period 1:  2016  N=39 | | Period 2:  2016-2017  N=122 | | Period 3:  2017-2018  N=205 | | P-value Chi^2^ for trend |
| --- | --- | --- | --- | --- | --- | --- | --- | --- |
|  |  | **n** | ***%*** | **n** | ***%*** | **n** | ***%*** |  |
| Region | Flanders | 8 | *20.5* | 70 | *57.4* | 119 | *58.1* | **<0.001** |
|  | Wallonia | 27 | *69.2* | 46 | *37.7* | 68 | *33.2* |  |
|  | Brussels | 4 | *10.3* | 6 | *4.9* | 18 | *8.8* |  |
| Age (months) | 6-12 | 14 | *35.9* | 57 | *48.3* | 92 | *44.9* | 0.716 |
|  | 13-24 | 19 | *48.7* | 46 | *39.0* | 89 | *43.4* |  |
|  | 25-30 | 6 | *15.4* | 15 | *12.7* | 24 | *11.7* |  |
| Gender | male | 20 | *51.3* | 64 | *54.2* | 102 | *49.8* | 0.740 |
| Preterm delivery | yes | 0 | *0.0* | 13 | *11.2* | 12 | *5.9* | **0.038** |
| Breastfeeding >6 months | yes | 7 | *18.0* | 26 | *22.4* | 48 | *23.4* | 0.755 |
| At least one parent smokes | yes | 15 | *38.5* | 29 | *25.0* | 59 | *28.8* | 0.273 |
| Previous hospitalisation | yes | 13 | *33.3* | 33 | *28.5* | 62 | *30.2* | 0.842 |
| Number of siblings | 0 | 3 | *7.7* | 30 | *26.1* | 84 | *41.0* | **<0.001** |
|  | 1 | 14 | *35.9* | 41 | *35.7* | 66 | *32.2* |  |
|  | >1 | 22 | *56.4* | 44 | *38.2* | 55 | *26.8* |  |
| AOM-history | 0 | 0 | *0.0* | 0 | *0.0* | 101 | *49.3* | **<0.001** |
|  | 1 | 13 | *52.0* | 22 | *41.5* | 51 | *24.9* |  |
|  | >1 | 12 | *48.0* | 31 | *58.5* | 53 | *25.9* |  |
| AB <3 months | yes | 11 | *28.2* | 27 | *23.5* | 43 | *21.0* | 0.588 |

Table 6: Demographic and clinical characteristics of children with AOM per period with Chi-square p-value for trend in Belgium in 2016-2018.

*AOM= acute otitis media, AB= antibiotics.*

**Appendix 2**

*Table 7: Carriage frequency of Streptococcus pneumoniae serotypes in the AOM child population and the healthy child population attending DCC per season in Belgium in 2016-2018.*

|  | AOM | | | DCC | | | |
| --- | --- | --- | --- | --- | --- | --- | --- |
|  | **Dominating serotypes** | **n** | ***%*** | **Dominating serotypes** | **n** | ***%*** |  |
| Spring 2016 | 23B | 4 | *14.3* | 23B | 93 | *18.6* |  |
|  | 11A | 4 | *14.3* | 23A | 49 | *9.8* |  |
|  | 35F | 2 | *7.1* | 11A | 40 | *8.0* |  |
|  | 15B | 2 | *7.1* | 15B | 33 | *6.6* |  |
|  | 15C | 2 | *7.1* | 15A | 31 | *6.2* |  |
| Winter 2016-2017 | 23B | 14 | *16.9* | 23B | 155 | *19.9* |  |
|  | 15B | 8 | *9.6* | 15B | 62 | *7.9* |  |
|  | 11A | 5 | *6.0* | 10A | 59 | *7.6* |  |
|  | 15C | 5 | *6.0* | 23A | 58 | *7.4* |  |
|  | 10A | 4 | *4.8* | 21 | 54 | *6.9* |  |
|  | 21 | 4 | *4.8* | 11A | 51 | *6.5* |  |
| Winter 2017-2018 | 23B | 15 | *9.6* | 23B | 96 | *13.9* |  |
|  | 6C | 13 | *8.3* | 19A | 61 | *8.8* |  |
|  | 11A | 11 | *7.0* | 11A | 55 | *8.0* |  |
|  | 35B | 11 | *7.0* | 23A | 50 | *7.2* |  |
|  | 19A | 10 | *6.4* | 21 | 49 | *7.1* |  |
|  | 15B | 10 | *6.4* | 6C | 46 | *6.7* |  |
|  | 10A | 10 | *6.4* | 15B | 45 | *6.5* |  |

Only the most frequent serotypes together accounting for ca. 50% of carriage per season are presented. AOM= acute otitis media, DCC= day care centre.

**Appendix 3**

|  | | General | | Spring 2016 | | Winter 2016-2017 | | Winter 2017-2018 | |
| --- | --- | --- | --- | --- | --- | --- | --- | --- | --- |
|  |  | **AOM**  **n *(%)*** | **DCC n *(%)*** | **AOM**  **n *(%)*** | **DCC**  **n *(%)*** | **AOM**  **n *(%)*** | **DCC**  **n *(%)*** | **AOM**  **n *(%)*** | **DCC**  **n *(%)*** |
| PCV7 VT prevalence (%) | **19F** | 7 (2.6) | 42 (2.1) | 1 (3.6) | 13 (2.6) | 2 (2.4) | 13 (1.7) | 4 (2.6) | 16 (2.3) |
|  | **23F** | 0 (0.0) | 3 (0.2) | 0 (0.0) | 3 (0.6) | 0 (0.0) | 0 (0.0) | 0 (0.0) | 0 (0.0) |
|  | **14** | 1 (0.4) | 5 (0.3) | 1 (3.6) | 4 (0.8) | 0 (0.0) | 1 (0.1) | 0 (0.0) | 0 (0.0) |
| PCV10 VT prevalence (%) | **1** | 0 (0.0) | 1 (0.1) | 0 (0.0) | 1 (0.2) | 0 (0.0) | 0 (0.0) | 0 (0.0) | 0 (0.0) |
| PCV13 VT prevalence (%) | **19A** | 13 (4.9) | 79 (4.0) | 0 (0.0) | 3 (0.6) | 3 (3.6) | 15 (1.9) | 10 (6.4) | 61 (8.8) |
|  | **3** | 7 (2.6) | 16 (0.8) | 0 (0.0) | 4 (0.8) | 4 (4.8) | 5 (0.6) | 3 (1.9) | 7 (1.0) |
|  | **6A** | 0 (0.0) | 2 (0.1) | 0 (0.0) | 2 (0.4) | 0 (0.0) | 0 (0.0) | 0 (0.0) | 0 (0.0) |
| NVT prevalence (%) | **23B** | 33 (12.3) | 344 (17.4) | 4 (14.3) | 93 (18.6) | 14 (16.9) | 155 (19.9) | 15 (9.6) | 96 (13.9) |
|  | **11A** | 20 (7.5) | 146 (7.4) | 4 (14.3) | 40 (8.0) | 5 (6.0) | 51 (6.5) | 11 (7.0) | 55 (8.0) |
|  | **15B** | 20 (7.5) | 140 (7.1) | 2 (7.1) | 33 (6.6) | 8 (9.6) | 62 (7.9) | 10 (6.4) | 45 (6.5) |
|  | **6C** | 17 (6.3) | 68 (3.5) | 1 (3.6) | 9 (1.8) | 3 (3.6) | 13 (1.7) | 13 (8.3) | 46 (6.7) |
|  | **10A** | 15 (5.6) | 111 (5.6) | 1 (3.6) | 27 (5.4) | 4 (4.8) | 59 (7.6) | 10 (6.4) | 25 (3.6) |
|  | **35B** | 15 (5.6) | 94 (4.8) | 1 (3.6) | 17 (3.4) | 3 (3.6) | 42 (5.4) | 11 (7.0) | 35 (5.1) |
|  | **35F** | 12 (4.5) | 73 (3.7) | 2 (7.1) | 14 (2.8) | 2 (2.4) | 35 (4.5) | 8 (5.1) | 24 (3.5) |
|  | **23A** | 11 (4.1) | 157 (8.0) | 1 (3.6) | 49 (9.8) | 3 (3.6) | 58 (7.4) | 7 (4.5) | 50 (7.2) |
|  | **21** | 11 (4.1) | 126 (6.4) | 0 (0.0) | 23 (4.6) | 4 (4.8) | 54 (6.9) | 7 (4.5) | 49 (7.1) |
|  | **15C** | 11 (4.1) | 78 (4.0) | 2 (7.1) | 18 (3.6) | 5 (6.0) | 36 (4.6) | 4 (2.6) | 24 (3.5) |
|  | **15A** | 10 (3.7) | 95 (4.8) | 1 (3.6) | 31 (6.2) | 2 (2.4) | 41 (5.3) | 7 (4.5) | 23 (3.3) |

*Table 8: Prevalence of S. pneumoniae serotypes overall and per season in Belgium in 2016-2018.*

AOM= acute otitis media, DCC= day care centre, PCV7= 7-valent pneumococcal conjugate vaccine PCV10= 10-valent pneumococcal conjugate vaccine, PCV13= 13-valent pneumococcal conjugate vaccine, NVT= non-vaccine serotype.

**Appendix 4**

Table 9: Clinical severity score in children with AOM per serotype group, Belgium 2016-2018. PCV7= 7-valent pneumococcal conjugate vaccine, PCV13= 13-valent pneumococcal conjugate vaccine

| capsulartype | Median clinical score |  | clinical score<6 | | clinical score >6 | | |
| --- | --- | --- | --- | --- | --- | --- | --- |
|  |  |  | **n** | ***%*** | **n** | ***%*** |  |
| Non-vaccine type | 6 |  | 100 | *43.7* | 129 | *56.3* |  |
| PCV7 serotype | 6 |  | 3 | *37.5* | 5 | *62.5* |  |
| PCV13 serotype | 5.5 |  | 10 | *50.0* | 10 | *50.0* |  |
| Untypeable | 5 |  | 2 | *50.0* | 2 | *50.0* |  |
